# Supplementary material for: Satisfaction and associated factors towards inpatient health care services among adult patients at Pawie General Hospital, West Ethiopia
Source: PLoS One. 2021 Apr 20;16(4):e0249168. doi: 10.1371/journal.pone.0249168 (PMC8057596; doi:10.1371/journal.pone.0249168)
Supplement: Attachment — Submitted filename: 12-Revised Manuscript with Track Changes_for PLoS.docx [file pone.0249168.s002.docx]

**Influence of number of individuals and observations per individual on an estimate of subgroup structure**

Julia Sunga^1^, Quinn M.R. Webber^2^, and Hugh Broders^1,3^*

^1^Department of Biology, University of Waterloo, Waterloo, Ontario, Canada

^2^ Cognitive and Behavioural Ecology Interdisciplinary Program, Memorial University of Newfoundland, St. John’s, Newfoundland and Labrador, Canada

^3^Former Address: Department of Biology, Saint Mary’s University, Halifax, Nova Scotia, Canada

*Corresponding author

Email: [hugh.broders@uwaterloo.ca](mailto:hugh.broders@uwaterloo.ca) (HB)

**Abstract**

Social network analysis is being increasingly relied upon to understand the ecology of animal groups. However, it is not always feasible to observe every relevant interaction among all individuals in a natural population. To date, studies have assessed how missing information affects estimates of an individual’s position in the network, but less attention has been paid to global network metrics which are fundamental to understanding the causative agents for a network’s structure. Further, in populations with highly connected subgroups, such as societies displaying fission-fusion dynamics, missing information may affect our understanding of global network structure differently than in societies with distinctly separated subgroups. Using a bat maternity group showing fission-fusion dynamics, we provide an example of the effects missing data can have on the ability to detect and quantify subgroup structure. In our system, fewer than 30 observations per individual, regardless of the number of individuals included, resulted in low reliability in estimates of the number of subgroups with less than 50% of iterations correctly estimating the actual number of subgroups present. However, in scenarios where >25% of the individuals are sampled, each with >10 observations, we were able to reliably detect that subgroups were present, not the number of subgroups, and appropriately assort individuals. We recommend that researchers exercise caution when making conclusions about animal social networks when a substantial number of individuals or observations are missing, and when possible, consider subsampling large datasets to observe how estimates are influenced by sampling intensity. We support that including more observations per individual should be prioritized over including more individuals when resources are limited. Our study serves as an example of the reliability, or lack thereof, of global network measures with missing information, and further work is needed to determine how sampling regime performance will vary with different data collection methods, network structures, sizes, and sampling periods.

**Keywords**

Community assortativity, fission-fusion dynamics, maternity colony, modularity, *Myotis lucifugus*, sample size, social network analysis, subgroups

**Introduction**

Social network analysis permits an in-depth understanding of population-level social structure and has been widely applied to study the organization of animal groups (Bejder et al. 1998; Whitehead 1999; Webber and Vander Wal 2019). Social groups may vary in size, composition, and structure, and large societies may show hierarchical organization, with higher order groups comprised of subgroups in which individuals associate more frequently within than among subgroups (Newman 2006; Aureli et al. 2012). These subgroups may not be static in structure, demonstrating fission-fusion dynamics as they merge and split through space and time (Aureli et al. 2008; Whitehead 2008; Sueur and Maire 2014). For fission-fusion societies, individuals can interact to varying extents with conspecifics from many subgroups, making the identification of subgroup boundaries difficult. Subgroup structure can still be delineated, when sufficient data exists, based on repeated or consistent associations among individuals. These consistencies in association, and where the associations occur, can provide insight into whether subgroup formation is the result of passive processes such as use of a common resource, or active processes such as social preference among conspecifics, among other factors (Bejder et al. 1998; Whitehead 1999; Croft et al. 2011; Papageorgiou et al. 2019; Grueter et al. 2020). Testing these types of hypotheses about social structure in societies with fission-fusion dynamics requires sufficient data on interactions among individuals to reliably assign subgroup membership (Webber and Vander Wal 2019).

A common challenge for studies using social network analysis, particularly in natural systems, is collecting enough data to reliably estimate social structure, and determining how much sampling is needed. Specifically, the proportion of a group, or total number of individuals sampled, and the number of observations per individual must be sufficient to reflect the dynamics of the true network. In a recent review of social network literature, Webber & Vander Wal (2019) identified the median number of individuals included per network was 15 (range 4–1406), but many studies did not indicate what proportion of the overall population this may represent. Further, the minimum number of observations included per individual varies widely among studies and can have important effects on network estimates. For example, based on assessment of individual network metric performance, Farine and Strandburg-Peshkin (2015) recommended that there should be at least 20 observational periods where a pair of individuals are observed either together or apart (see also Franks et al., 2010; Hoppitt & Farine, 2018; Lusseau et al., 2008; Silk et al., 2015; Whitehead, 2008). Other studies select a median number of observations per individual as a decision rule (i.e., Mourier et al., 2012), which, in well sampled populations, may unnecessarily omit individuals or pairs of individuals that have fewer observations than the designated threshold. In contrast, in poorly sampled populations, the median number of observations per individual may retain individuals that do not have sufficient information with which to estimate their network position. Generally, increasing the minimum number of observations required per individual reduces the number of individuals that can be included in an analysis, effectively decreasing the estimated network size and increasing the proportion of the population that is analytically considered as ‘missing’ (Silk 2018).

Multiple studies have assessed the effect of missing data on the estimation of animal social networks, largely focusing on impacts on estimates of an individual’s position in the network (e.g., degree, centrality) (Frantz et al. 2009; Franks et al. 2010; Silk et al. 2015; Hoppitt and Farine 2018). Specific thresholds can vary based on the network metrics of interest and the type of network, but important broad patterns relating to the effect of missing data are consistent across most studies. Across all network sizes, studies have suggested minimums of 10-50% of the population sampled and unsurprisingly, the more observations that can be obtained of these individuals, the better (Franks et al. 2010; Silk et al. 2015). However, Davis et al. (2018) also note that in some systems, increased sampling intensity may not be a priority because networks become saturated, such that estimates do not improve with further sampling unless all individuals or interactions can be observed. Importantly, requirements of a minimum number of observations per dyad or median threshold should vary with the type and timing of the social system being studied (Frantz et al. 2009), and the behaviour or interaction of interest (Davis et al. 2018). Thus, recommendations may vary among different social systems (e.g., fission-fusion or multilevel dynamics). It has also been recommended to more intensely sample a smaller proportion of the population when resources are limited (Franks et al. 2010; Silk et al. 2015). However, for fission-fusion societies, the potential for ephemeral social connections, which can impact the functions and consequences of animal societies (e.g., information transfer and disease transmission; Granovetter, 1973), differs from societies with more stable group structure. Meanwhile, too few observations per individual may lead to spurious estimates of the strength of connections among pairs of individuals leading to inaccurate estimates of network structure and further work is needed to determine how these recommendations may vary across different types of social societies. We therefore expect a need to balance minimum requirements for observations to accurately assess individual connections, while also including as many individuals as possible to model a larger proportion of the overall population.

When assessing a new system, various strategies can be applied to determine the robustness of estimates to missing data, and to determine what amount of data is needed for a desired level of confidence. The simplest to apply of these is bootstrapping, where aspects of the network are repeatedly assessed with varying degrees of missing data (Franks et al. 2010; Shizuka and Farine 2016). This technique is integral to the community assortativity estimate process described by Shizuka and Farine (2016) and has also been applied to assess the robustness of network measures (Franks et al. 2010; Silk et al. 2015). Further, a variation of bootstrapping, jackknifing, in which data is systematically removed, is commonly used to assess the error associated with estimates of lagged associate rates (Whitehead 2008). Bayesian methods can also be applied to assess the uncertainty around edge weight estimates (Farine and Strandburg-Peshkin 2015).

Here, we use bootstrapping to examine the impact of varying numbers of observations per individual and number of individuals included in social network analysis on estimates of global network structure in a fission-fusion society. Generally, it is expected that the inclusion of more information in a sample decreases the discrepancy between the estimate and the true network structure, but that reasonable estimates could be obtained with substantial amounts of missing data. Using an empirical dataset of a large fission-fusion social network of little brown myotis (*Myotis lucifugus*), we quantified the impacts of two distinct sampling considerations on estimates of subgroup structure. Specifically, we assessed how numbers of individuals and observations per individual affected the ability of a sub-sample of the network to detect the presence of subgroups, estimate the number of subgroups, and reliably assort individuals into subgroups in this type of system.

**Materials and methods**

**Sample Population**

We used data from a long-term study of wild *Myotis lucifugus* roosting in artificial structures at Salmonier Nature Park, Newfoundland, Canada in 2016. Individuals were implanted with passive integrated transponder (PIT) tags and readers (Trovan, Ltd. United Kingdom) were deployed at 11 roost boxes within a ≈1 km^2^ area of the park, each of which were regularly used throughout the study period. Other natural and anthropogenic roosts existed in the area that were not monitored. Readers recorded observations of when individuals entered and exited roost boxes and the last observation before sunrise constituted the daytime roost locations of individual bats. Dyadic associations were based on the gambit-of-the-group assumption (Whitehead 2008), such that individuals were assumed to associate if they roosted in the same box on the same day. It is known that there were missing observations and that at least 50% of individuals in the population were not tagged nor included in this study. This dataset does not necessarily represent the complete population however characterization of the actual true population is not the purpose of this study. Rather, our aim is to quantify differences in estimates between samples where all information is included, to those with only partial data.

All animal handling protocol was approved by the animal care committee of Saint Mary’s University, Halifax, Nova Scotia (AUP #16-12). Wildlife scientific research permits were also obtained from the Government of Newfoundland and Labrador, Department of Fisheries and Land Resources, Forestry and Wildlife Branch (# WLR2016-12).

**Generating the ‘observed’ network**

All detections were filtered for adult female bats that were each observed on ≥ 40 days, which resulted in 99 individuals. For each individual, 40 of their observations, day records of an individual bat in a particular roost, were randomly selected to generate a balanced dataset (all individuals observed an equal number of times, albeit not on the same days) from which we calculated an ‘observed’ social network that spanned 131 observation days. These numbers were selected to provide more observations per individual than has been assessed in other studies, such as Franks et al. 2010 which assessed reliability up to a maximum of 20 censuses, while still allowing multiple levels of sub-setting of the number of individuals included. Although a network with a maximum of 40 observations removes additional information on individuals in an already incomplete dataset, we used this threshold to ensure that remove any mpact of individuals with uneven numbers of available observations. Further, the ‘observed’ network was not assumed to be a reliable estimate of the true underlying population, and as such all findings and implications focus on discrepancies between the observed network and the sampled networks derived from the observed network.

From our subset of data, we calculated a network based on the instances of co-roosting, which represent individuals observed in the same roost on the same day. We first calculated the Half Weight Index (HWI, Whitehead 2008) between all dyads based on the proportion of days spent roosting together or apart using package “asnipe” (Farine 2013). The HWI represented edges in the network. Next, we calculated modularity (Q) using the “igraph” package (Farine, 2016) to estimate the number of subgroups, a process which iteratively divides the network and measures the ratio of the density of connections within and among subgroups until a maximal value is reached. We then assessed the confidence in the division of the network both by the detection of subgroups and the confident assortment of individuals into subgroups. First, 1000 random networks were generated using “spatsoc” (Robitaille et al. 2019) to randomly swap individuals among roosts on a given day, while maintaining the number of available boxes and individuals constant within days, generating a distribution of Q values. A p-value was calculated, via Monte Carlo methods, based on the percent of random network Q values that fell above that of the original focal network, where a p-value of < 0.05 indicated significant evidence for subgroup structure. This measure may only support an inference that there is evidence for subgroup structure and does not provide confidence in the estimated number of subgroups or the assignment of individuals to these subgroups. The second metric we tested, as proposed by Shizuka and Farine (2016), examined the confidence in the assignment of individuals to subgroups by calculating a community assortativity coefficient (R_com_). The R_com_ method uses bootstrapping of a focal network to calculate consistency in individual community assignment, resulting in a network-level value between -1 and 1. Values closer to 1 indicate high confidence in subgroup assortment while values closer to 0 indicate little confidence in subgroup assortment. Shizuka and Farine (2016) recommend a threshold of 0.5 for reasonable confidence in subgroup assortment. Calculation of R_com_ via bootstrapping was repeated 10 times to obtain an average value for the ‘observed’ network.

**Sampled networks**

In this study, we assessed the influence of two distinct sampling considerations, the number of individuals included, and the number of observations included per individual. All sample networks were based on randomly selecting a subset of individuals and observations from the above generated ‘observed’ network. Individuals were randomly sampled to generate subsets of 5, 10, 15, 20, 25, 35, 50, 75, and 99 (all) individuals. Within each of these network sizes, we randomly selected 1, 5, 15, 10, 20, 30, or 40 (all available from ‘observed’ network) observations for each individual to generate balanced sample networks. This sampling process was repeated 100 times for each combination of number of individuals and number of observations per individual. A sample network of 99 individuals with 40 observations per individual was not created as this would represent the complete ‘observed’ network.

For all sample networks, the number of subgroups based on Q was estimated as above for the ‘observed’ network. P-values were calculated by generating 1000 random networks for each replicate and comparing the distribution of Q values via Monte Carlo methods as above. These random networks shuffled the roost an individual bat roosted in on a particular day, and were generated with “spatsoc” (Robitaille et al. 2019). Those with a p-value < 0.05, indicating significant evidence for subgroups structure were assigned a YES (1), while those with a p-value > 0.05 were assigned a NO (0). R_com_ values were also calculated as outlined by Shizuka and Farine (2016). The number of bootstraps was set equal to the number of individuals in the sample network. On occasion, particularly in networks with only 1 observation per individual, no connections between individuals were present in the sample network, resulting in a Q value of zero and returning a network where the number of detected subgroups was equal to the number of individuals present. This scenario does not constitute subgroup structure and because there was no ability to assign individuals to subgroups, the subgrouping value was coded as a NO (0) while R_com_ values were set to 0.

**Statistical Analyses**

To assess how sampling regime affected estimates of subgroup structure, the absolute value of the difference in estimated number of subgroups between the ‘observed’ network and the sample networks was calculated. Multiple linear regression was used to assess whether the independent variables of the number of observations, the number of individuals, and the interaction between these factors influenced the difference between the sample and ‘observed’ network in estimated number of subgroups. Both response and independent variables were log-transformed to meet model assumptions of normality and heteroscedasticity of residuals. For each sampling regime, the proportion of iterations that estimated the number of subgroups exactly was also calculated.

Often, network analysis simply aims to determine whether there is sufficient evidence of subgroup structure in a study population (e.g. Garroway & Broders, 2007). A logistic regression was performed on whether there was evidence for subgroup structure (p < 0.05) in the sample networks as there was in the ‘observed’ network. Number of individuals, number of observations per individual, and their interaction were used as the independent variables and the response variable was whether there was significant evidence for subgroup structure (1), or insufficient evidence (0; p > 0.05).

We also tested how different sampling regimes affected the ability to confidently assort individuals based on community assortativity (R_com_ > 0.5; Shizuka & Farine, 2016). As other assortativity measures, measures of how strongly nodes associate with those most like themselves, have been reported to be artificially high in poorly sampled systems (Franks et al. 2010), a linearly increasing R_com_ value was not expected with the addition of more information. We therefore tested the probability of obtaining a R_com_ estimate that indicated confidence in individual assortment (R_com_ ≥ 0.5), without overestimating this confidence by exceeding the value of the ‘observed’ network. Values within this range were coded as acceptable (1) while those above or below this range as unacceptable (0). A logistic regression was then performed with this binary response variable and the number of observations, number of individuals, and the interaction between these factors as independent variables. All sampling, randomizations, network metric calculation, and statistical analysis were performed in R version 3.6.0 (R Core Team, 2013).

**Results**

**Relationship between sampling regime and estimated number of subgroups**

The ‘observed’ network of 99 individuals with 40 observations per individual had 3 subgroups with significant evidence for subgroup structure (Q = 0.268, p < 0.001) and a R_com_ of 0.855, indicating a high-level of confidence in the assortment of individuals into subgroups. Across all sample networks, the estimated number of subgroups ranged from 2 to 94 (median = 4, SE ± 0.176). Multiple linear regression revealed a significant relationship between the number of individuals (β = 1.03, SE = 0.01, p < 0.001), number of observations per individual (β = 0.298, SE= 0.02, p < 0.001), and the interaction between these terms (β = -0.368, SE= 0.005, p < 0.001), on the discrepancy between the ‘observed’ number of subgroups and the number estimated by sample networks (p < 0.001, R^2^ = 0.866). Generally, error associated with our estimates was large when the number of observations was low (< 10) and increased when more individuals were included with few (< 10) observations (Fig 1). Regardless of the number of individuals included, the number of subgroups was more regularly estimated to be between 2 to 4 (within one of the observed network) when at least 15 observations per individual (94.3%) were included compared to when 10 or fewer observations were included (11.9%) in the analysis.

**Fig 1. Variation in the estimated number of subgroups under various scenarios of number of individuals sampled and the number of observations per individual (panels A-H).** Point sizes are scaled based on the number of times a value occurred. The red horizontal line represents the actual number of subgroups in the ‘observed’ population from which all other scenarios were sampled (subgroups = 3). Note, the changing y-axis scale among panels.

**Relationship between sampling regime and evidence for subgroup structure**

Analysis of the p-values of Q indicated a significant increase in the probability of correctly concluding the presence of subgroup structure with additional individuals (β = 6.00 x 10^-3^, SE = 1.7 x 10^-3^ p < 0.01) and observations (β = 5.25 x 10^-2^, SE = 4.7 x 10^-3^, p < 0.001), and an interactive effect between these variables (β = 4.03 x 10^-3^, SE = 2.2 x 10^-4^, p < 0.001). With < 10 observations per individual, the probability of correctly concluding the presence of subgroup structure did not exceed 75% even with all individuals included. With ≥ 10 observations per individual, the probability of detecting subgroup structure exceeded 50% with < 30 individuals sampled (Fig 2A).

**Fig 2. Probability of detecting subgroup structure (A) and confident assortment of individuals (B)**. Detection of subgroups (A) is based on the p-value of modularity (Q; p < 0.05) and confident assortment of individuals (B) is based on obtaining a community assortativity coefficient (R_com_) value that indicates a reasonable ability to assort individuals into subgroups (≥ 0.5) without overestimating this ability based on the ‘observed’ network (≤ 0.855).

R_com_ values ranged from 0 to 1 (median = 0.66, SE ± 0.004) across all sampling regimes. We observed a significant relationship between the probability of obtaining a R_com_ value indicating reasonable ability to assort individuals into subgroups (0.5 < R_com_ < 0.855) and the independent variables of number of individuals (β = 9.58 x 10^-3^, SE = 1.4 x 10^-3^, p < 0.001) and number of observations per individual (β = 4.39 x 10^-2^, SE = 3.4 x 10^-3^, p < 0.001; Fig 2B). The interaction term between the two independent variables was also significant (β = 7.55 x 10^-4^, SE = 9.4 x 10^-5^, p < 0.001). With 10 observations or fewer, R_com_ was overestimated relative to the observed network in 32.5% of replicates. With 15 or more observations per individual, estimates of R_com_ fell within the acceptable range in 66.4% of replicates regardless of the number of individuals included (Fig 3).

**Fig 3. Distribution of community assortativity coefficients (R_com_) as a factor of the number of individuals and the number of observations (panels A-H) included in the network.** Boxes represent the 25^th^ to 75^th^ percentile of values with the center line representing the median value. The blue line indicates the suggested minimum threshold of R_com_ (0.5) and the red line indicates the R_com_ value of the ‘observed’ network (0.855). Values that fell within this range were deemed acceptable and therefore represent sample networks with reasonable confidence in the assortment of individuals into subgroups.

**Summary of sampling regime performance**

At least 10 observations per individual were required to detect the presence of subgroup structure in at least 50% of replicates using the p value of Q (Table 1). More observations (15+) per individual were needed to obtain a R_com_ value above 0.5 but less than the ‘observed’ network value of 0.855 in at least 50% of replicates. The proportion of R_com_ estimates that fell within this range occasionally decreased with more observations per individual due to an increase in overestimates (Fig 3E-H). Across all numbers of individuals, the confidence in estimates of the number of subgroups exceeded 50% only when 20 or more observations per individual (of the 40 possible) were available and was at or near 0 with less than 10 observations for all numbers of individuals.

**Table 1. All sampling regimes (number of individuals and number of observations per individual) where there was sufficient evidence to detect subgroup structure in at least half of all 100 replicates.**

| Number of Individuals | Number of Observations | Number of replicates with sufficient evidence for subgroup structure (p<0.05) | Number of replicates with R_com_ >0.5 & <0.855 | Number of replicates that estimate 3 subgroups |
| --- | --- | --- | --- | --- |
| 5 | 40 | 51 | 17 | 13 |
| 10 | 20 | 58 | 62 | 65 |
| 10 | 30 | 82 | 57 | 54 |
| 10 | 40 | 83 | 52 | 47 |
| 15 | 15 | 57 | 73 | 41 |
| 15 | 20 | 72 | 64 | 59 |
| 15 | 30 | 88 | 69 | 60 |
| 15 | 40 | 98 | 68 | 66 |
| 20 | 15 | 60 | 68 | 35 |
| 20 | 20 | 80 | 77 | 47 |
| 20 | 30 | 97 | 63 | 69 |
| 20 | 40 | 99 | 59 | 69 |
| 25 | 10 | 56 | 80 | 6 |
| 25 | 15 | 72 | 64 | 25 |
| 25 | 20 | 89 | 75 | 47 |
| 25 | 30 | 97 | 70 | 64 |
| 25 | 40 | 100 | 58 | 70 |
| 35 | 15 | 87 | 75 | 34 |
| 35 | 20 | 98 | 83 | 48 |
| 35 | 30 | 100 | 76 | 64 |
| 35 | 40 | 100 | 65 | 65 |
| 50 | 10 | 69 | 33 | 0 |
| 50 | 15 | 99 | 67 | 28 |
| 50 | 20 | 100 | 93 | 49 |
| 50 | 30 | 100 | 73 | 58 |
| 50 | 40 | 100 | 70 | 71 |
| 75 | 10 | 90 | 38 | 2 |
| 75 | 15 | 100 | 93 | 35 |
| 75 | 20 | 100 | 97 | 52 |
| 75 | 30 | 100 | 79 | 63 |
| 75 | 40 | 100 | 58 | 80 |
| 99 | 10 | 99 | 49 | 2 |
| 99 | 15 | 100 | 96 | 19 |
| 99 | 20 | 100 | 98 | 52 |
| 99 | 30 | 100 | 93 | 94 |

Sufficient evidence for subgroup structure was based on a p < 0.05 when comparing values of Q to that of randomized networks. The number of replicates (0-100) with a community assortativity (R_com_) value considered acceptable at greater than the minimum threshold of 0.5 but less than the ‘observed’ network (0.855) are reported and the number of replicates (0-100) that correctly estimated the presence of 3 subgroups.

**Discussion**

In most studies of animal behaviour, it is recommended to include as much information as possible to estimate characteristics with the greatest probability of accuracy and to ensure reliable inference. Social network analysis is no exception, particularly when the gambit-of-the-group method is applied to deducing social connections (Franks et al. 2010). However, in ecological studies, sampling is often limited by factors such as logistical challenge and cost. We quantified the effects of both the number of individuals and the number of observations per individual on the reliability of metrics that characterize the subgroup structure in a fission-fusion social network created using the gambit-of-the-group assumption. As expected, in our system the ability to detect subgroup structure, assort individuals into subgroups, and estimate the number of subgroups in the network improved with the inclusion of more data but inclusion of more individuals and more observations did not have equal impacts on these different estimates. Thus, when designing studies of animal behaviour, there can be considerable trade-offs between these two sampling considerations and the metrics of interest.

Our results are consistent with prior studies based on estimates of individual network position (Silk et al. 2015; Hoppitt and Farine 2018) that recommended only including individuals for which a minimum number of observations have been recorded, and that adding more individuals cannot make up for a lack of repeated observations of individuals or interactions (Carter et al. 2019). We found that the presence of subgroups and confidence in individual assortment into subgroups could often be estimated with missing data but, in our dataset, 20 or more observations per individual were needed to consistently achieve an estimate within 2 of the ‘observed’ number of subgroups present, regardless of the number of individuals present. Additionally, including a larger number of individuals with less than 10 observations per individual greatly increased the average discrepancy between sample estimates and the ‘observed’ network. As these results demonstrate, including many individuals with insufficient information per individual has the potential to result in increased error when estimating group-level metrics. Yet, the use of overly conservative minimum observations thresholds when estimating network properties may reduce our ability to detect weak connections, which can be essential for understanding the overall network structure (Granovetter 1973). It is therefore important to balance including as many individuals as possible, while ensuring that all included individuals have been sampled sufficiently.

To select a sampling regime with sufficient potential to estimate animal social networks, we recommend that other studies with sufficiently large data sets perform similar subsampling tests to assess how their estimates behave in response to missing data and provide some suggestion of how requirements may change based on study objectives. For most studies interested in aspects of global network structure, the first priority is to determine whether the network shows evidence of any subgroup structure. In our ‘sample’ populations, conclusions consistent with the ‘observed’ population regarding the presence of subgroup structure could be made with as few as 10 observations and less than 50% of the population sampled, when using Monte Carlo methods to obtain a p-value for Q. The number of observations per individual had a strong impact on the ability to detect subgroup structure, as networks with < 10 observations with any number of individuals were not reliable and could not detect the presence of subgroups in more than 50 % of replicates. These findings support the recommendations of Franks et al. (2010), who suggested that when resources are limited, researchers should prioritize sampling fewer individuals more intensely rather than sampling more individuals less intensely and support that network metrics can be robust to missing individuals when enough observations are provided (Frantz et al. 2009). However, these results do not provide information on whether the estimated number of subgroups is accurate, or how well the estimated subgroups assort individuals.

To accurately estimate the number of subgroups present in this social network, far more information, and therefore a more intense sampling regime, was required than was needed to simply detect that subgroups were present. Overall, our ability to accurately estimate the number of subgroups was limited. Success for any particular sampling regime did not exceed 80% with the exception of having 30 observations per individual and 100% of individuals included, a feat that may be unreasonable for many ecological studies. Estimates of the number of subgroups did generally improve with the inclusion of more individuals or more observations per individuals but it was again important that at least 10 observations per individual were included. With 20-30 observations, the correct number of subgroups was estimated over 50% of the time, even with only 10 individuals included. These findings are again in agreement with the recommendation of Franks et al. (2010) to ensure a sufficient number of observations per individual. Given the densely overlapping nature of subgroups in fission-fusion societies, it is possible that estimates of subgroup numbers would perform better in less highly connected networks with the same sampling regime (Frantz et al. 2009; Ding et al. 2016).

Silk et al. (2015) suggested that global measures of social networks would perform poorly when individuals are missing from a network. The ability to provide significant evidence for the presence of subgroup structure based on p-values of Q and the modest ability to estimate the number of subgroups present despite missing individuals, provided sufficient numbers of observations shown here contradicts this proposition. This may be due to the fact that other global metrics, such as clustering coefficient, can be greatly influenced by the omission of single individuals that bridge two groups or subgroups (Lee et al. 2006; Silk et al. 2015). Our results suggest that when selecting a social network metric to determine the presence of subgroups for populations that are under-sampled, Q may be a robust choice, however further work is needed to ensure that the performance of Q is not unique to our specific network structure. Further, the performance of global metrics may vary based on the type of sampling used in a study, and the definition of edges in a network (Davis et al. 2018).

Regardless of the number of subgroups, the ability to consistently assort individuals to subgroups is useful for understanding network structure and asking questions about mechanisms of group and subgroup formation. R_com_ provides an opportunity to estimate the confidence in the assortment of individuals to specific subgroups (Shizuka and Farine 2016). Generally, our analysis of R_com_ demonstrated the performance of assigning individuals to groups was more variable than detecting subgroups via p-values of Q, but more consistent than estimating the exact number of subgroups within any given sampling regime. There was a tendency to overestimate the R_com_, a similar result to other assortativity measures, namely assortment into groups by sex or other traits of the individuals (Franks et al. 2010). It is unsurprising that the R_com_ may be vulnerable to the same issues. Specifically, we found that R_com_ was often overestimated when individuals were observed < 10 times. This may be due to less variation between bootstrap removals of individuals when networks contained fewer observations. As the number of observations increased beyond 20 per individual, we found that R_com_ stabilized, a result which is corroborated by Franks et al. (2010) and emphasizes the benefit of increasing the sampling intensity for each individual.

Unlike other studies on the effects of missing information on network estimates, we do not suggest any specific thresholds for estimation of global network metrics as our results may not be suitable to all networks. Specifically, our ‘observed’ network has only a small number of subgroups, and it is expected that with more subgroups, estimates from subsamples would be more variable, and the ability to assort individuals in these subgroups would decline. Thus, more subdivided networks may require a larger proportion of the population to be sampled, and/or more observations per individual to appropriately characterize the network subgroup structure. Further, the difference in average strength of connections within and among subgroups may be more variable in other networks. In networks where subgroups are less connected, such as those of other bat species, *Thyroptera tricolor* (Vonhof et al. 2004), fewer observations per individual may be appropriate to delineate subgroups. Important considerations for differences in the rate of interactions and variability in network structure will also impact sampling protocols (Davis et al. 2018). Finally, our ‘observed’ network does not contain all individuals and interactions that occur in the population, and thus it is unknown how well our ‘observed’ network represents the true, underlying population of bats in our study area. This topic would benefit from further study on how global network estimates preform when the underlying population is known with more certainty, for example, using captive populations that are continuously monitored.

As our network serves as only an example of how estimates of global network metrics may vary in a specific type of fission-fusion system, we recommend that, as these types of larger datasets become more readily available, researchers continue to assess how subsampling and therefore missing data influences network estimates. There may be additional benefits to testing how subgroup detection, R_com_, and the estimated number of subgroups vary with network size, as the effect of sampling effort on the accuracy of other network measures has been found previously to vary with overall network size (Silk et al. 2015). Given that our ‘observed’ network of 99 tagged individuals was much smaller than what is expected in the true population, and in some other social networks assessed to date (exceeding 1000 individuals; Webber & Vander Wal, 2019), predictive ability may be higher in actual bat social networks when the same proportions of the population are observed. Another potential avenue of future research is to assess how R_com_ can be adapted for less stable animal social societies, including those of bats. It is likely that increasing connectivity among subgroups despite high density connections within subgroups, as is typical in fission-fusion societies, negatively influences our ability to correctly assort individuals to a subgroup. A modification of R_com_ has been suggested for instances where an individual is a part of multiple subgroups simultaneously (Montilla et al. 2019), and perhaps a similar modification is needed for societies displaying fission-fusion dynamics. Further study is necessary to determine how both network size and structure can influence the consequences of sampling and data filtering prior to generating network models based on a portion of the overall population (Frantz et al. 2009; Silk et al. 2015).

The temporal extent of data collection also has the potential to influence social network structure and assignment of individuals to subgroups. Specifically, our data were collected over a relatively long period of time (131 days) representing the full range of dates when at least one animal was observed in the study area. This results in the multiple observations of an individual not necessarily occurring on the same days as observations for other individuals. Although, it is likely that days where individuals were not observed occurred when individuals were not present in the system and therefore not associating with the observed animals. Always observing individuals synchronously, as is the case in many animal network study systems, is expected to improve network estimates and possibly result in fewer observations per individual being required to achieve comparable levels of reliability (Davis et al. 2018). A shorter, more heavily sampled time period should provide increased confidence in R_com_ and estimates of the number of subgroups but, shorter time-periods represent only a snapshot of the social environment. Networks are inherently dynamic systems and inferences across too narrow a temporal window may overestimate the strength of relationships between individuals and subgroups or miss important transient connections (Blonder et al. 2012; Pinter-Wollman et al. 2014; Zhao et al. 2018; Bonnell and Vilette 2020). Further, obtaining 20 observations but over a very long time-period would likely be insufficient as more samples would be needed to appropriately reflect such a dynamic system throughout the entire study period. Thus, when designing studies, researchers must carefully consider the temporal period of network formation that is biologically relevant for their study species (e.g. reproductive period or breeding season), and the expected rate of interactions or behaviour of interest (Davis et al. 2018) and select an appropriate sampling regime for the designated period.

**Conclusions**

Overall, our study suggests that the detection of subgroup structure via Q and appropriate assortment of individuals into subgroups can be robust to missing information, while estimating the exact number of subgroups is less reliable. To determine what level of sampling is required, researchers must consider their study system, sampling design, and decide which inferences are most important and what level of precision or reliability is desired. When designing a study, results in our system support the suggestion of Franks et al. (2010) to prioritize sampling a smaller part of the population with greater intensity when resources are limited, rather than more of the population at lower intensity to improve network estimates. The levels of reliability that we present here reflect that of a highly connected, fission-fusion society and results may differ for different network sizes and types (Frantz et al. 2009). Generally, we recommended that researchers include as many individuals and observations as is feasible, but we urge caution when including individuals with limited numbers of observations. Our results represent preliminary background for selecting a sampling regime and continued work is needed to determine how the effects of both missing individuals and missing observations varies with different types of animal societies. As large datasets such as ours become increasingly available, further work should continue to investigate how missing data effects the reliability of estimates of global network metrics. When it is uncertain whether enough individuals or observations have been collected, there may be benefit to subsampling and calculating network metrics, as we have here, to determine if estimates stabilize as the full sample size is approached.

**Authors’ contributions**

JS and HB conceived of the study and general methodology, HB provided the initial dataset, JS developed the statistical methods, conducted all analyses with support from QMRW. All authors contributed critically to development of the drafts and gave final approval for submission.

**Acknowledgements**

We would like to acknowledge the contributions of the many hands that were involved in data collection over the many years of monitoring including Karissa Appleby, Nicole Besler, Michael Blackwood, Lynne Burns, Mackenzie Donahue, Cody Fouts, Amanda Gallant, Alicia Irwin, Laura Johnson, Shelley Moores, Jordi Segers, and Kayley Smith. We would also like to acknowledge the important contributions of Jessica Humber, Bruce Rodrigues, and the entire Salmonier Nature Park Staff over the last eight years, and many other Department of Environment and Conservation Staff who have participated.

**References**

Aureli, F., Schaffner, C.M., Asensio, N., and Lusseau, D. 2012. What is a subgroup? How socioecological factors influence interindividual distance. Behav. Ecol. **23**(6): 1308–1315. doi:10.1093/beheco/ars122.

Aureli, F., Schaffner, C.M., Boesch, C., Bearder, S.K., Call, J., Chapman, C.A., Connor, R., Fiore, A. Di, Dunbar, R.I.M., Henzi, S.P., Holekamp, K., Korstjens, A.H., Layton, R., Lee, P., Lehmann, J., Manson, J.H., Ramos‐Fernandez, G., Strier, K.B., and Schaik, C.P. van. 2008. Fission‐Fusion Dynamics. Curr. Anthropol. **49**(4): 627–654. doi:10.1086/586708.

Bejder, L., Fletcher, D., and Brager, S. 1998. A method for testing association patterns of social animals. Anim. Behav. **56**: 719–725.

Blonder, B., Wey, T.W., Dornhaus, A., James, R., and Sih, A. 2012. Temporal dynamics and network analysis. Methods Ecol. Evol. **3**(6): 958–972. doi:10.1111/j.2041-210X.2012.00236.x.

Bonnell, T.R., and Vilette, C. 2020. Constructing and analysing time-aggregated networks: The role of bootstrapping, permutation and simulation. Methods Ecol. Evol. (00): 1–13. doi:10.1111/2041-210X.13351.

Carter, G.G., Schino, G., and Farine, D. 2019. Challenges in assessing the roles of nepotism and reciprocity in cooperation networks. Anim. Behav. **150**: 255–271. Elsevier Ltd. doi:10.1016/j.anbehav.2019.01.006.

Croft, D.P., Madden, J.R., Franks, D.W., and James, R. 2011. Hypothesis testing in animal social networks. Trends Ecol. Evol. **26**(10): 502–507. doi:10.1016/j.tree.2011.05.012.

Davis, G.H., Crofoot, M.C., and Farine, D.R. 2018. Estimating the robustness and uncertainty of animal social networks using different observational methods. Anim. Behav. **141**: 29–44. Elsevier Ltd. doi:10.1016/j.anbehav.2018.04.012.

Ding, Z., Zhang, X., Sun, D., and Luo, B. 2016. Overlapping Community Detection based on Network Decomposition. Sci. Rep. **6**(April): 1–11. Nature Publishing Group. doi:10.1038/srep24115.

Farine, D.R. 2013. Animal social network inference and permutations for ecologists in R using asnipe. Methods Ecol. Evol. **4**(12): 1187–1194. doi:10.1111/2041-210X.12121.

Farine, D.R. 2016. Package ‘ assortnet ’: Calculate the Assortativity Coefficient of Weighted and Binary Networks.

Farine, D.R., and Strandburg-Peshkin, A. 2015. Estimating uncertainty and reliability of social network data using Bayesian inference. R. Soc. Open Sci. **2**(9): 1–13. doi:10.1098/rsos.150367.

Franks, D.W., Ruxton, G.D., and James, R. 2010. Sampling animal association networks with the gambit of the group. Behav. Ecol. Sociobiol. **64**(3): 493–503. doi:10.1007/s00265-009-0865-8.

Frantz, T.L., Cataldo, M., and Carley, K.M. 2009. Robustness of centrality measures under uncertainty: Examining the role of network topology. Comput. Math. Organ. Theory **15**(4): 303–328. doi:10.1007/s10588-009-9063-5.

Garroway, C.J., and Broders, H.G. 2007. Nonrandom association patterns at northern long-eared bat maternity roosts. Can. J. Zool. **85**(9): 956–964. doi:10.1139/Z07-079.

Granovetter, M.S. 1973. The Strength of Weak Ties. Am. J. Sociol. **78**(6): 1360–1380.

Grueter, C.C., Qi, X., Zinner, D., Bergman, T., Li, M., Xiang, Z., Zhu, P., Migliano, A.B., Miller, A., Krützen, M., Fischer, J., Rubenstein, D.I., Vidya, T.N.C., Li, B., Cantor, M., and Swedell, L. 2020. Multilevel Organisation of Animal Sociality. Trends Ecol. Evol. **35**(9): 834–847. Elsevier Ltd. doi:10.1016/j.tree.2020.05.003.

Hoppitt, W.J.E., and Farine, D.R. 2018. Association indices for quantifying social relationships: how to deal with missing observations of individuals or groups. Anim. Behav. doi:10.1016/j.anbehav.2017.08.029.

Lee, S.H., Kim, P.J., and Jeong, H. 2006. Statistical properties of sampled networks. Phys. Rev. E - Stat. Nonlinear, Soft Matter Phys. **73**(1): 1–7. doi:10.1103/PhysRevE.73.016102.

Lusseau, D., Whitehead, H., and Gero, S. 2008. Incorporating uncertainty into the study of animal social networks. Anim. Behav. **75**(5): 1809–1815. doi:10.1016/j.anbehav.2007.10.029.

Montilla, L.M., Ascanio, A., Verde, A., and Croquer, A. 2019. Systematic review and meta-analysis of 50 years of coral disease research visualized through the scope of network theory. PeerJ **7**: e7041. doi:10.7717/peerj.7041.

Mourier, J., Vercelloni, J., and Planes, S. 2012. Evidence of social communities in a spatially structured network of a free-ranging shark species. Anim. Behav. **83**(2): 389–401. Elsevier Ltd. doi:10.1016/j.anbehav.2011.11.008.

Newman, M.E.J. 2006. Modularity and community structure in networks. PNAS **103**(23): 8577–8582. doi:https://doi.org/10.1073/pnas.0601602103.

Papageorgiou, D., Christensen, C., Gall, G.E.C., Klarevas-Irby, J.A., Nyaguthii, B., Couzin, I.D., and Farine, D.R. 2019. The multilevel society of a small-brained bird. Curr. Biol. **29**(21): R1120–R1121. Elsevier. doi:10.1016/j.cub.2019.09.072.

Pinter-Wollman, N., Hobson, E.A., Smith, J.E., Edelman, A.J., Shizuka, D., De Silva, S., Waters, J.S., Prager, S.D., Sasaki, T., Wittemyer, G., Fewell, J., and McDonald, D.B. 2014. The dynamics of animal social networks: Analytical, conceptual, and theoretical advances. Behav. Ecol. **25**(2): 242–255. doi:10.1093/beheco/art047.

Robitaille, A.L., Webber, Q.M.R., and Vander Wal, E. 2019. Conducting social network analysis with animal telemetry data: Applications and methods using spatsoc. Methods Ecol. Evol. **10**(8): 1203–1211. doi:10.1111/2041-210X.13215.

Shizuka, D., and Farine, D.R. 2016. Measuring the robustness of network community structure using assortativity. Anim. Behav. **112**: 237–246. doi:10.1016/j.anbehav.2015.12.007.

Silk, M.J. 2018. The next steps in the study of missing individuals in networks: a comment on Smith et al. (2017). Soc. Networks **52**: 37–41. Elsevier B.V. doi:10.1016/j.socnet.2017.05.002.

Silk, M.J., Jackson, A.L., Croft, D.P., Colhoun, K., and Bearhop, S. 2015. The consequences of unidentifiable individuals for the analysis of an animal social network. Anim. Behav. **104**: 1–11. Elsevier Ltd. doi:10.1016/j.anbehav.2015.03.005.

Sueur, C., and Maire, A. 2014. Modelling animal group fission using social network dynamics. PLoS One **9**(5): e97813. doi:10.1371/journal.pone.0097813.

Vonhof, M.J., Whitehead, H., and Fenton, M.B. 2004. Analysis of Spix’s disc-winged bat association patterns and roosting home ranges reveal a novel social structure among bats. Anim. Behav. **68**(3): 507–521. doi:10.1016/j.anbehav.2003.08.025.

Webber, Q.M.R., and Vander Wal, E. 2019. Trends and perspectives on the use of animal social network analysis in behavioural ecology: a bibliometric approach. Anim. Behav. **149**: 77–87. Elsevier Ltd. doi:10.1016/j.anbehav.2019.01.010.

Whitehead, H. 1999. Testing association patterns of social animals. Anim. Behav. **57**: 26–29.

Whitehead, H. 2008. Analyzing Animal Societies: Quantitative Methods for Vertebrate Social Analysis. University of Chicago Press, Chicago, USA.

Zhao, M.J., Driscoll, A.R., Sengupta, S., Stevens, N.T., Fricker, R.D., and Woodall, W.H. 2018. The effect of temporal aggregation level in social network monitoring. PLoS One **13**(12): e0209075. doi:10.1371/journal.pone.0209075.
